# Empathy among Saudi Residents at a Tertiary Academic Center during the COVID-19 Pandemic and Its Association with Perceived Stress

**DOI:** 10.3390/medicina58091258

**Published:** 2022-09-11

**Authors:** Haytham I. AlSaif, Mamdouh N. Alenezi, Mohammed Asiri, Khalid O. Alshaibani, Abdullah A. Alrasheed, Saad M. Alsaad, Mohammed A. Batais

**Affiliations:** 1 Department of Family and Community Medicine, College of Medicine, King Saud University, P.O. Box 2925 (34), Riyadh 11461, Saudi Arabia; 2 Department of Medicine, College of Medicine, King Saud University, P.O. Box 2925 (38), Riyadh 11461, Saudi Arabia; 3 Tufts Medical Center, Psychiatry, Boston, MA 02111, USA

**Keywords:** empathy, psychological stress, residency, COVID-19, Saudi Arabia

## Abstract

*Background and Objectives*: Empathy is an important attribute of a healthy doctor–patient relationship. Although multiple studies have assessed empathy in different countries, little is known about its levels among Saudi residents and its association with perceived stress. Objectives: To assess the levels of empathy and to identify if there is an association with stress in general and across the demographic and training characteristics of residents. *Materials and Methods*: A cross-sectional questionnaire-based study was carried out from December 2020 to March 2021 among residents training at a tertiary academic center in Riyadh, Saudi Arabia. Empathy and perceived stress were measured using the Jefferson Scale of Empathy (JSE) and the Perceived Stress Scale (PSS). *Results*: A total of 229 residents participated. The mean JSE score was 105.25 ± 15.35. The mean JSE scores were significantly higher among residents training in pediatrics (mean difference (MD) = 17.35, *p* < 0.001), family medicine (MD = 12.24, *p* = 0.007), and medical specialties (MD = 11.11, *p* = 0.012) when compared with surgical specialties and anesthesia. In addition, residents who worked 1–4 on-calls per month had a higher mean JSE score (MD = 11.23, *p* = 0.028) compared with those who worked 7 or more on-calls. Lastly, no correlation between empathy and perceived stress was detected in the whole sample (r = −0.007, *p* = 0.913); however, there was a correlation among residents training in medical specialties (r = −0.245, *p* = 0.025). *Conclusion*: Residents in our study had empathy levels comparable with Asian but lower than Western residents. We recommend qualitative studies that explore potential factors that might affect empathy among residents and studying the association between empathy and perceived stress among medical residents. Postgraduate curricula should incorporate interventions that foster a more empathetic doctor–patient relationship.

## 1. Introduction

Empathy is defined as “a predominantly cognitive (rather than an affective or emotional) attribute that involves an understanding (rather than feeling) of experiences, concerns and perspectives of the patient, combined with a capacity to communicate this understanding, and an intention to help” [1]. Physicians’ empathy has been linked with multiple benefits, such as increased patient satisfaction, better clinical outcomes, and lower rates of complications [2,3]. Residents were found to have lower levels of empathy when they were compared with both first- and second-year medical students and specialists [4,5]. A systematic review that included longitudinal studies conducted on residents in the USA showed a progressive decline in empathy throughout residency years [6]. A later cross-sectional study from the USA showed lower empathy scores among second- and fourth-year residents compared with first-year residents [7]. However, a study conducted in Singapore did not find a difference in empathy levels between residency years [8], and a study from South Korea found higher empathy among fourth-year residents compared with first-year residents [9]. Previous research showed that medical students who had higher empathy were more likely to choose the following specialties for residency training: internal medicine, family medicine, obstetrics and gynecology (ob/gyn), pediatrics, and psychiatry [10]. This finding was replicated in studies measuring empathy levels among physicians including residents in training [1,9]. Generally, previous research on medical students and physicians showed higher empathy scores among females [1]. Considering the studies conducted only on residents, some found higher levels of empathy among females [9,11]; however, other studies did not find any difference between genders [4,7,12,13].

Perceived stress is a common mental health issue among residents in training [14,15]. Residents can face different types of stressors: training-related, work-related, social, and financial [14]. Furthermore, the burden of the COVID-19 pandemic might have exaggerated the effect of previous stressors and be a stressor itself with the concern of acquiring the infection or transmitting it to family members [16]. The association between stress and empathy has previously been studied mainly in medical students and pediatric residents, with conflicting results between positive, negative, or no correlations [17,18,19,20,21]. Authors attributed positive correlations to the fact that students suffering from stress might become more empathetic with others who were suffering, such as patients [18]. Interestingly, Michalec et al. suggested that students might adapt to stress during medical school by becoming less empathetic to decrease their vulnerability to stress [17]. Studies that incorporated interventions to decrease stress among residents reported improvement in empathy levels [22,23,24,25,26,27,28]. Notably, when constructs of empathy were considered, mindfulness-based stress reduction (MBSR) resulted in a significant improvement in perspective taking among residents [24].

Perceived stress, especially occupational stress, has been associated with burnout in general and with the emotional exhaustion and depersonalization constructs of burnout in medical residents [20,29,30,31,32]. On the other hand, multiple studies have been conducted on residents to study the association between empathy and burnout [5,8,9,30,31,33,34,35,36,37]. Some of the studies reported an association between higher empathy and lower overall burnout [5,30,32,37]. Conversely, other studies did not find a similar association [33,35,36]. Looking more closely at the associations between empathy and the constructs of burnout [5,8,9,31,33,34,35], most studies reported a negative correlation between empathy and depersonalization [5,8,9,31]. However, Olson et al. did not find a correlation between empathy and depersonalization [33], and, interestingly, Lafreniere et al. found a positive association between residents’ depersonalization and patients’ ratings of their empathy [34]. Nevertheless, when the association between the emotional exhaustion construct of burnout and empathy in residents was studied, mixed results were reported. For instance, Park et al. and Lee et al. reported a negative correlation [8,9], while Olson et al. and Huang et al. did not find any correlation [31,33].

When empathy levels among medical students from Kingdom of Saudi Arabia (KSA) and Kuwait were assessed, they were found to be lower than their counterparts in Western countries yet similar to medical students from Asian countries [18,38,39]. Abdulkader et al. studied empathy among a small sample (*n* = 30) of physicians from a single center in Riyadh, KSA; however, they did not report whether they included residents in training or not [40]. Meanwhile, studies that describe levels of empathy among residents in KSA are lacking. In addition, there are few studies that investigated the association between perceived stress and empathy among residents. Therefore, we sought to assess empathy levels among trainees enrolled at residency programs in an academic tertiary medical center in KSA. Furthermore, we aimed to determine if there were associations between empathy levels and perceived stress, stressors, and concern about COVID-19 infection or transmission to residents’ families.

## 2. Methods

### 2.1. Study Design and Setting

This was a cross-sectional, online survey that included residents training at King Saud University Medical City (KSUMC), Riyadh, KSA, during the period from December 2020 to March 2021. The KSUMC is a tertiary academic medical city that comprises three hospitals (King Khalid University Hospital, King Abdulaziz University Hospital, and Dental University Hospital).

### 2.2. Eligibility Criteria and Classification of Specialties

In the present study, eligible participants included residents training in any academic year at KSUMC. Residents were allowed to participate if they were enrolled in clinical specialties that require physical contact with patients. Therefore, residents enrolled in the following residency programs were excluded: anatomical histopathology, hematopathology, medical microbiology, medical biochemistry, pathology, and preventive medicine. Furthermore, interns (i.e., last-year medical students in KSA), dental and pharmacy residents, and fellows were excluded from participation. Ultimately, we obtained a complete list of 597 residents who were eligible for participation from the Postgraduate Medical Education Center at King Saud University. Residents were classified by specialty based on the curricula of their training rotations. Internal medicine and its subspecialties (dermatology, neurology, and psychiatry) were classified as one category (medical), general surgery, anesthesia, and surgical subspecialties (cardiac surgery, neurosurgery, ophthalmology, orthopedics, otorhinolaryngology, pediatric surgery, plastic surgery, urology) as one category (surgical and anesthesia), and emergency medicine and critical care as one category. Lastly, family medicine, ob/gyn, pediatrics, and radiology were kept as they were.

### 2.3. Research Instruments and Data Collection

We developed a self-administered questionnaire in English using the SurveyMonkey^®^ (Momentive, San Mateo, CA, USA) online platform. The questionnaire comprised 6 parts and 40 items: (1) sociodemographic and residency-related characteristics (five items), including gender, marital status, residency year, specialty, and the specialty in which the resident is currently working; (2) workload characteristics (three items), including the number of on-calls or shifts during the past month and the perceived difference between current workload (during the COVID-19 pandemic) and usual workload before the pandemic; (3) a multiple-response item on the possible stressors (work-related, financial, or social); (4) two Likert-scale items on concern about acquiring COVID-19 and transmitting the infection to family members; (5) the 10-item Perceived Stress Scale (PSS-10) [41]; (6) and the 20-item Jefferson Scale of Empathy (JSE) health professions version [1]. A pilot study was initially conducted with 17 residents (who were not included in the final analysis) to calculate the mean JSE score and to obtain feedback from participants specifically about the ease of access, comprehension, and length of the questionnaire. There were no issues with access or comprehension; however, participants reported that the questionnaire was lengthy; thus, we opted to use the 4-item PSS instead of the 10-item questionnaire [42]. JSE-Health Professions consists of 20 items answered on a 7-point Likert scale. The JSE measures 3 main constructs of empathy, including perspective taking (10 items), compassionate care (8 items), and standing in the patient’s shoes (2 items). The responses were coded from 1 (strongly disagree) to 7 (strongly agree). Ten items were positively worded, and the remaining items were negatively worded. The coding of the negatively worded items was reversed, and a total JSE score was calculated for each participant (ranging from 20 to 140), where higher JSE scores indicated greater empathy [1]. On the other hand, PSS-4 consists of 4 items that measure perceived stress in the past month on a 5-point Likert scale. Responses to items 1 and 4 were coded from 0 (never) to 4 (very often), while items 2 and 3 were reverse-coded. The total score ranged between 0 and 16, and higher scores indicated higher stress levels [41,42].

### 2.4. Sample Size and Participants’ Recruitment

We calculated the sample size required to detect a significant difference from the results of Lee et al. (mean JSE score 104.9 ± 13.2) utilizing G*Power software version 3.1 (Faul et al., Düsseldorf, Germany). We considered an expected mean JSE score of 101.5 (calculated from our pilot study), a power of 95%, and an α of 0.05 to detect a significant difference. The result was a sample size of 198 participants.

We prepared a message that contained the study rationale and objectives as well as a direct link to the consent form and study questionnaire. This message was shared with residents through their chief resident in their social media groups used for training-related announcements. Lastly, they were reminded of participation twice on a monthly basis.

### 2.5. Ethical Considerations

Permission to use the JSE-Health Professions was obtained from Thomas Jefferson University (Philadelphia, PA, USA), which allows up to 3 out of the 20 items to be illustrated. PSS 4 and 10 do not require permission to use. Ethical approval was obtained from the institutional review board (IRB) of the College of Medicine at King Saud University (project number: E-20–4998). The objectives of the study were explained to all respondents, and they provided consent prior to their participation. Participation was voluntary, and no self-identifying information was mentioned in the list obtained nor collected in the questionnaire, including name, identification number, phone number, and email. Data were only collected for research purposes and stored on the password-protected computer of the principal investigator.

### 2.6. Statistical Analysis

Statistical analysis was carried out using the Statistical Package for Social Sciences version 26.0 (IBM Corporation, Armonk, NY, USA). Questionnaires with missing responses to PSS-4 or JSE were excluded. Categorical variables were expressed as frequencies and percentages, whereas means, medians, interquartile ranges (IQR), and standard deviations (SDs) were used to present continuous variables. Cronbach’s alpha was used to measure the internal consistency of PSS-4 and JSE. The differences in PSS-4 and JSE scores across sociodemographic and residency-related characteristics were assessed using a *t*-test for two independent groups (gender and the marital status) and one-way analysis of variance (ANOVA) for variables with three or more groups. Tukey or Games–Howell post hoc tests were performed on all pairwise multiple comparisons for significantly different groups. Correlations between PSS-4 and JSE scores and continuous or ordinal variables were tested using Pearson’s or Spearman’s correlation tests, respectively. Statistical significance was considered at *p* < 0.05.

## 3. Results

### 3.1. Sociodemographic, Residency-Related, and Workload Characteristics

The number of respondents was 252 residents, which comprises 127.3% of the sample size calculated and 42.2% of the total target population. However, we excluded one participant because his specialty was pathology and 22 participants due to missing primary outcomes (responses to the PSS-4 or JSE scales). Therefore, 229 questionnaires were analyzed. The highest response rate was among family medicine residents (91.5%), followed by medical (50%), pediatrics (45.3%), emergency medicine and critical care (35.9%), ob/gyn (31.6%), surgical (15.3%), and radiology (11.4%). The sample comprised 115 males (50.2%) and 162 single participants (70.7%). Most respondents were enrolled in the following residency programs: medical (32.8%), family medicine (23.6%), and pediatrics (14.8%). During the preceding month, 73.0% of respondents were working on-calls, with 45% working 1–4 on-calls per month. Approximately one-third of the participants (29.7%) indicated that their workload in the preceding month was higher than their usual pre-COVID-19 pandemic workload. The distribution of the sociodemographic, residency-related, and workload characteristics of participating residents is shown in Table 1.

### 3.2. Sources of Stress

**Figure 1:**
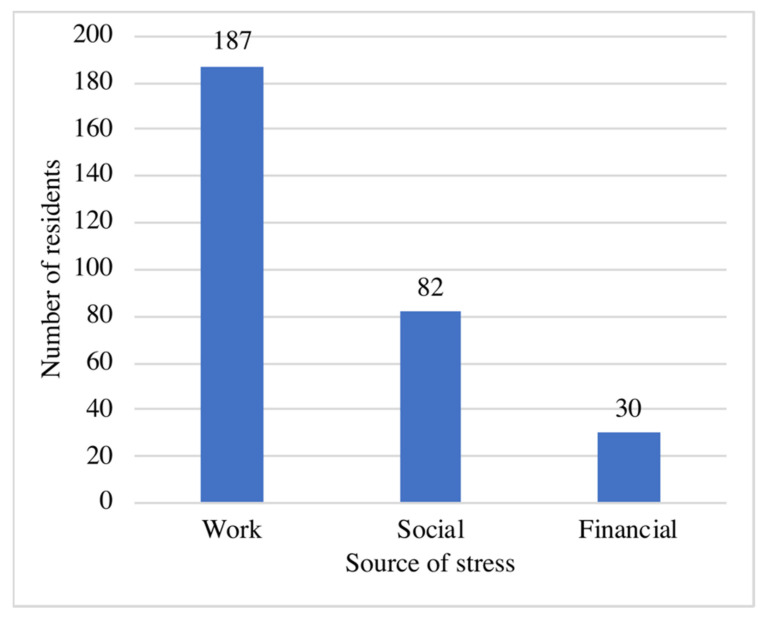
Frequency of sources of stress reported by the residents (*n* = 200).

**Figure 2:**
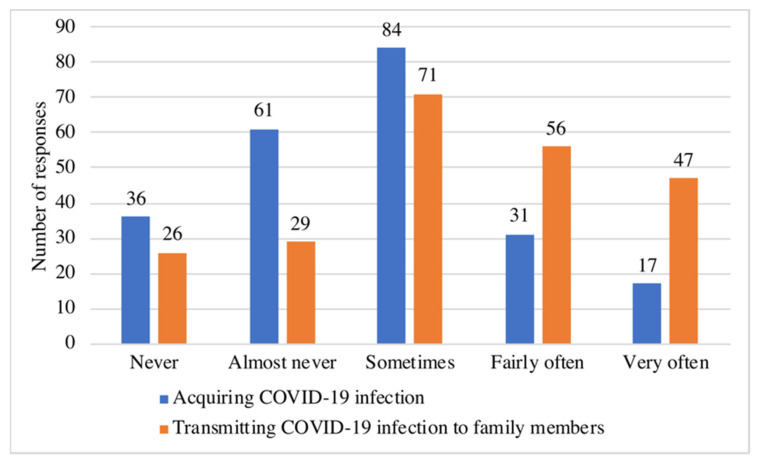
Residents’ responses regarding their concerns about COVID-19 infection (*n* = 229).

In general, 29 (12.7%) residents reported that they did not have any particular source of stress. On the other hand, the remaining 200 participants selected 296 multiple responses from the available 3 sources of stress. The highest reported source of stress was work-related stress, which was reported by 187 residents (81.7%). Figure 1 illustrates the frequency of sources of stress reported by the residents. Regarding the risk of COVID-19 infection, residents were more concerned about the possibility of transmitting the infection (responding “very often” or “fairly often”) to their families (45.0%) than about acquiring the infection themselves (21%). Figure 2 displays residents’ responses regarding their concerns about COVID-19 infection.

### 3.3. Perceived Stress Scale

The Cronbach’s alpha for the PSS-4 was 0.652. The median (IQR) PSS-4 score of all the residents was 7.0 (5.0–9.0). Females and residents who reported having work or social sources of stress had significantly higher mean PSS-4 scores. Moreover, there were medium and small positive correlations between the PSS-4 score and the number of sources of stress reported by the resident and the concerns about both acquiring COVID-19 and transmitting it to family members, respectively. The mean PSS-4 scores and correlation coefficients across the sociodemographic, residency-related, and workload characteristics of residents are shown in Table 2.

### 3.4. Jefferson Scale of Empathy

**Figure 3:**
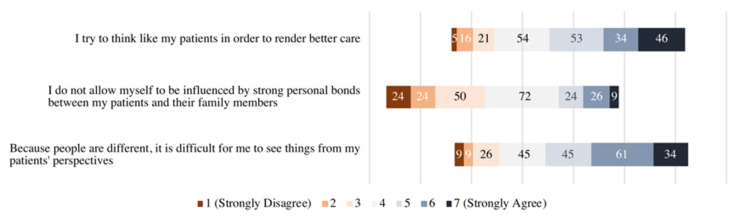
Stacked bar chart of residents’ responses to items 6, 17, and 18 of the JSE (*n* = 229).

The Cronbach’s alpha for the JSE scale was 0.839. The median (IQR) JSE score of the whole sample was 106.0 (95.0–117.0). The mean scores for the perspective taking, compassionate care, and standing in the patient’s shoes constructs of the JSE were 53.78 ± 10.08, 41.37 ± 7.02, and 10.09 ± 2.48, respectively. The lowest mean scores for an item from the standing in patient’s shoes, perspective taking, and compassionate care constructs were for items 6 (4.86, out of 7), 17 (4.83), and 18 (3.71), respectively. Figure 3 shows a stacked bar chart of residents’ responses to the aforementioned items. The mean JSE score was higher among females compared with males, but the difference was not statistically significant. However, empathy mean scores differed significantly within the categories of residents’ specialty, residency level, and residents’ number of on-calls in the preceding month. Mean scores and correlation coefficients of JSE across the sociodemographic, residency-related, and workload characteristics of residents are shown in Table 2. Subsequently, a post hoc multiple-comparisons test revealed a significant mean JSE score difference between medical and surgical and anesthesia residents, family medicine and surgical and anesthesia, pediatrics and emergency and critical care, radiology, and surgical and anesthesia residents. In addition, fourth-year residents had significantly higher mean JSE scores than fifth-year residents, and residents working one to four on-calls per month had higher JSE score than residents who worked seven or more on-calls. The results of significant post hoc analyses are summarized in Table 3, and the detailed post hoc analyses are supplemented in Appendix A (Table A1, Table A2, Table A3 and Table A4).

### 3.5. Correlation between Empathy and Stress

In the whole sample, PSS-4 score did not correlate with the JSE score (r = −0.007, *p* = 0.913) or with its constructs: perspective taking (r = −0.003, *p* = 0.96), compassionate care (r = −0.037, *p* = 0.568), and standing in patient’s shoes (r = 0.076, *p* = 0.248). There was a small, nonsignificant, negative correlation (r = −0.146, *p* = 0.119) between JSE and PSS-4 scores among male residents. There was a small, significant, negative correlation (r = −0.245, *p* = 0.025) between JSE and PSS-4 scores among residents training in a medical specialty at the time of participation. In addition, there were nonsignificant, moderate positive (r = 0.386, *p* = 0.126) and negative (r = −0.366, *p* = 0.373) correlations among residents rotating in ob/gyn and radiology, respectively. Table 4 shows the correlation coefficients across the sociodemographic, residency-related, and workload characteristics of residents.

## 4. Discussion

The mean empathy score among residents in our study (105.25) was similar to scores previously reported among medical students (106.55 and 105.18) but lower than that among physicians (111) in KSA [38,39,40]. On the other hand, we did not find a difference in empathy scores between residency years. The only exception was between fourth-year and fifth-year residents, which can be explained by the fact that we had a few (*n* = 5) fifth-year residents from specialties with low empathy scores, such as surgical and ob/gyn. When taking together, the fact that empathy scores were similar between our study and previous studies of medical students in KSA and that there were no differences between residency years in empathy levels suggests that empathy does not decline in the transition following medical school or throughout the residency years. Although a systematic review of longitudinal studies and a cross-sectional study from the USA suggest a decline in empathy during clinical training [6,7], this was not the case in cross-sectional Singaporean and South Korean studies [8,9]. Therefore, there is a need for a longitudinal study to investigate the changes in empathy among Saudi residents.

When compared with studies conducted among residents in different parts of the world, the mean empathy scores in our study were higher than that for Iranian residents (100.6) [43], generated mixed results compared with Asian countries (104.6, similar to Singapore; higher than South Korea, 93.59; and lower than Japan, 114.46) [8,9,36] and lower than residents in European countries, the USA, and Latin America (111.8–119.1) [5,11,12,13,44,45,46]. Previous research indicated multiple contributors to these differences in empathy levels: first, medical education factors such as the selection of residents and nature of training [47]; second, cultural and social influences on the doctor–patient relationship [1,9]; third, work-related factors such as working hours and on-calls [8,29]; and fourth, study-related factors such as the sampling and timing of data collection. We believe that qualitative studies on residents in KSA will be useful for exploring the factors that negatively affect residents’ empathy.

Females in our study had higher levels of empathy; however, and similar to previous studies [4,7,12,13], the difference was small and did not reach statistical significance. It is not surprising that residents from pediatrics, family medicine, and other medical specialties obtained the highest empathy scores [1,9,10]. On the other hand, and also consistent with the literature, residents from surgical specialties, anesthesia, and radiology achieved the lowest scores [9,10,44]. Interestingly, residents training in emergency medicine and critical care had low scores for empathy. This is inconsistent with previous research [1,9]. Residents in these specialties usually work in shifts, which might partially contribute to their low scores since residents who worked in shifts in our study had lower empathy scores, although these were not statically significant given the small number of shiftwork groups (*n* = 21). Another potential explanation is that the doctor–patient relationship in emergency medicine and critical care lacks continuity compared with other specialties [4]. Lastly, Passalacqua et al. reported lower empathy levels among residents at the end of the on-calls compared with the start [29]. This might explain why residents who worked seven or more on-calls per month had significantly lower empathy levels than their counterparts with one to four on-calls per month.

We did not find a significant correlation between perceived stress and empathy or its three constructs, including perspective taking. This is similar to previous studies conducted with medical students and pediatric residents that found no associations [17,20,21]. It is possible that the conflicting results of correlation between empathy and stress in different specialties might explain why we did not find a correlation between empathy and stress in the sample as a whole. For instance, the correlation between empathy and stress was negative among residents from medical specialties, while it was positive among ob/gyn residents. Furthermore, these conflicting results between specialties calls for further studies on each individual specialty. The only exception was among residents who were training in medical specialties at the time of participation in this study. Medical rotation is characterized by providing care to adult patients with advanced illness and multiple comorbidities in addition to demanding on-calls that provide little time to sleep or rest [48,49]. There were multiple subgroups with small-to-moderate correlations between stress and empathy that did not reach statistical significance due to relatively small group numbers, such as male residents and residents from the ob/gyn specialty. Those subgroups could be targets of further research investigating the association between perceived stress and empathy. Other items on stress in our study—such as having a work-related or social source of stress, number of stress sources, and COVID-19-related concerns—showed significant associations with PSS-4. However, they did not show any association with empathy.

The present study utilized validated questionnaires for the assessment of stress and empathy. This is the first study that investigated the levels of empathy specifically among Saudi residents in training programs. In addition, it adds to the literature on the association between empathy and perceived stress. This study was conducted during the second wave of the COVID-19 pandemic (Winter 2021), and it reflects the levels of empathy shown during those circumstances.

## 5. Limitations

This study was carried out in a single training center and used convenience sampling, which might limit the generalizability of the results. The use of a short scale (PSS-4) might have affected the measurement of perceived stress. Due to its cross-sectional design, the study collected data at a single point in time, which might have limited the ability to establish temporal relationships. Lastly, participants from certain specialties (for example radiology) were underrepresented due to low response rates.

## 6. Conclusions

Residents in our study had empathy levels comparable with those of residents from Asian countries but lower than those of residents from Western countries. We did not find a difference between residency years with regard to empathy levels. Subgroups with lower empathy included residents training in surgical specialties and anesthesia, emergency and critical care, and radiology and residents who worked seven or more on-calls in the past month. There was no correlation between empathy and perceived stress as a whole, but there were correlations within certain subgroups. We recommend the following for further research: longitudinal studies to investigate if there are changes in empathy during training; qualitative studies to explore potential factors that might affect empathy among residents, including on-calls; and studies on the association between empathy and perceived stress among male physicians; and residents training in medical and ob/gyn specialties. Lastly, postgraduate medical education curricula should incorporate interventions that foster a more empathetic doctor–patient relationship.

## Figures and Tables

**Table 1:** Sociodemographic, residency-related, and workload characteristics of participating residents (*n* = 229).

Variable	Category	Frequency	Percentage
Gender	Female	114	49.8
	Male	115	50.2
Marital status	Married	67	29.3
	Single	162	70.7
Specialty	Emergency and critical care	23	10
	Family medicine	54	23.6
	Medical	75	32.8
	Obstetrics and gynecology (ob/gyn)	12	5.2
	Pediatrics	34	14.8
	Radiology	4	1.7
	Surgical and anesthesia	27	11.8
Current rotation	Emergency and critical care	30	13.1
	Family medicine	20	8.7
	Medical	83	36.2
	Pediatrics	17	7.4
	Ob/Gyn	33	14.4
	Radiology	8	3.5
	Surgical and anesthesia	37	16.2
	Research	1	0.4
Residency Year	R1	58	25.3
	R2	66	28.8
	R3	43	18.8
	R4	57	24.9
	R5	5	2.2
Number of on-calls in the previous month	No on-calls	41	17.9
	1–4	103	45.0
	5–6	48	21.0
	7 or more	16	7.0
Shift work	Yes	21	9.2
	No	208	90.8
Workload during COVID-19 pandemic compared to usual workload	Less than usual	78	34.1
	Unaffected	83	36.2
	More than usual	68	29.7

**Table 2:** Mean scores and correlation coefficients of PSS-4 and JSE scores across the sociodemographic, residency-related, and workload characteristics of residents (*n* = 229).

Variable	Category	PSS-4		JSE	
		Mean ± SD	*p* Value ^†^	Mean ± SD	*p* Value ^†^
Scores	Whole sample	7.37 ± 2.72	NA	105.25 ± 15.35	NA
Gender	Female	8.13 ± 2.7	<0.001	107.20 ± 14.83	0.055
	Male	6.61 ± 2.53		103.31 ± 15.67	
Marital status	Married	7.06 ± 2.7	0.272	106.40 ± 16.05	0.466
	Single	7.49 ± 2.72		104.77 ± 15.08	
Specialty	Emergency and critical care	7.09 ± 3.16	0.501	97.83 ± 10.38	<0.001
	Family medicine	7.07 ± 2.49		108.09 ± 13.18	
	Medical	7.2 ± 2.64		106.96 ± 14.37	
	Ob/Gyn	8.17 ± 3.35		100.58 ± 19.41	
	Pediatrics	8.18 ± 2.53		113.21 ± 14.09	
	Radiology	7.75 ± 2.22		87.25 ± 7.18	
	Surgical and anesthesia	7.22 ± 2.97		95.85 ± 17.54	
Current rotation	Emergency and critical care	6.70 ± 2.97	0.154	102.93 ± 14.58	0.002
	Family medicine	7.15 ± 2.58		107.70 ± 12.97	
	Medical	7.20 ± 2.54		105.96 ± 14.05	
	Ob/Gyn	8.18 ± 3.03		99.47 ± 16.96	
	Pediatrics	8.45 ± 2.56		114.15 ± 13.27	
	Radiology	7.25 ± 2.19		100.75 ± 18.11	
	Surgical and anesthesia	7.11 ± 2.93		99.92 ± 17.40	
Residency year	R1	7.69 ± 2.72	0.743	103.62 ± 14.17	0.032
	R2	7.5 ± 2.79		105.7 ± 15.80	
	R3	7.14 ± 2.24		104.79 ± 13.67	
	R4	7.07 ± 2.88		108.37 ± 15.72	
	R5	7.2 ± 4.02		80.6 ± 21.7	
		**Spearman’s** **Correlation**	***p* value**	**Spearman’s** **Correlation**	***p* value**
		−0.088	0.185	0.087	0.189
Number of on-calls in the previous month	No on-calls	6.88 ± 2.54	0.065	104.68 ± 13.62	0.042
	1–4	7.1 ± 2.72		107.8 ± 14.47	
	5–6	8.17 ± 2.75		105.1 ± 16.56	
	7 or more	7.88 ± 2.31		96.56 ± 15.23	
Shift work in the previous month	Yes	7.43 ± 3	0.913	100.81 ± 17.71	0.165
	No	7.36 ± 2.7		105.7 ± 15.07	
Workload during the past month compared to pre-pandemic workload	Less than usual	7.15 ± 2.6	0.5	102.72 ± 17.61	0.2
	Unaffected	7.31 ± 3.1		106.64 ± 14.08	
	More than usual	7.67 ± 2.29		106.46 ± 13.83	
Source of stress					
Work	Yes	7.78 ± 2.65	<0.001	104.84 ± 15.6	0.396
	No	5.55 ± 2.25		107.07 ± 14.22	
Social	Yes	8.37 ± 2.66	<0.001	107.41 ± 13.62	0.111
	No	6.81 ± 2.6		104.04 ± 16.15	
Financial	Yes	7.67 ± 2.77	0.518	105.23 ± 12.35	0.995
	No	7.32 ± 2.71		105.25 ± 15.78	
Number of sources of stress	0	4.86 ± 2.01	<0.001	106.48 ± 13.94	0.419
	1	7.29 ± 2.51		103.73 ± 16.84	
	2	8.43 ± 2.49		107.57 ± 13.89	
	3	8.12 ± 3.24		104.82 ± 11.44	
		**Spearman’s** **Correlation**	***p* value**	**Spearman’s** **Correlation**	***p* value**
		0.343	<0.001	0.023	0.733
The concern of acquiring COVID-19 infection	Never	6.39 ± 2.36		109.14 ± 15.12	0.371
	Almost never	7.56 ± 3.07	0.02	104.34 ± 15.27	
	Sometimes	7.12 ± 2.64		103.43 ± 15.53	
	Fairly often	8.45 ± 2.41		106 ±12.77	
	Very often	8 ± 2.35		107.88 ± 19.04	
		**Spearman’s** **Correlation**	***p* value**	**Spearman’s** **Correlation**	***p* value**
		0.165	0.012	−0.01	0.879
The concern of transmitting COVID-19 to family members	Never	5.96 ± 2.13	0.024	105.23 ± 15.79	0.986
	Almost never	7.52 ± 2.73		104.97 ± 16.31	
	Sometimes	7.11 ± 2.79		104.62 ± 14.88	
	Fairly often	7.77 ± 3.07		105.30 ± 15.11	
	Very often	7.96 ± 2.17		106.32 ± 16.08	
		**Spearman’s** **Correlation**	***p* value**	**Spearman’s** **Correlation**	***p* value**
		0.191	0.004	0.054	0.417

**^†^***t* test or one-way analysis of variance (ANOVA).

**Table 3:** Pairwise comparisons with significant differences in means between categories based on post hoc analysis.*

Variable	Group 1	Group 2	Difference between Groups’ Means	Std. Error	*p* Value ^†^
Specialty	Medical	Surgical and anesthesia	11.11	3.22	0.012
	Family medicine	Surgical and anesthesia	12.24	3.38	0.007
	Pediatrics	Emergency and critical care	15.38	3.87	0.002
		Radiology	25.96	7.58	0.013
		Surgical and anesthesia	17.35	3.70	<0.001
Current rotation	Pediatrics	Emergency and critical care	11.22	3.76	0.048
		Ob/Gyn	14.68	4.44	0.019
		Surgical and anesthesia	14.23	3.56	0.002
Residency year	R4	R5	21.77	7.05	0.019
Number of on-calls in the past month	1–4	7 or more	11.23	4	0.028

* Detailed post hoc analyses are supplemented in Appendix A. **^†^** Tukey’s multiple-comparisons test.

**Table 4:** Correlations between PSS-4 and JSE scores for the whole sample and across sociodemographic and training characteristics (*n* = 229).

Variable	Category	PSS-4 and JSE	
		Pearson’s Correlation	*p* Value
Whole sample		−0.007	0.913
Gender	Male	−0.146	0.119
	Female	0.055	0.556
Marital status	Single	−0.06	0.446
	Married	0.127	0.305
Specialty	Emergency and critical care	0.044	0.839
	Family medicine	0.019	0.893
	Medical	−0.18	0.121
	Ob/Gyn	0.448	0.144
	Pediatrics	0.037	0.834
	Radiology	0.256	0.744
	Surgical and anesthesia	−0.125	0.534
Current rotation	Emergency and critical care	−0.018	0.925
	Family medicine	0.140	0.557
	Medical	−0.245	0.025
	Ob/Gyn	0.386	0.126
	Pediatrics	0.062	0.731
	Radiology	−0.366	0.373
	Surgical and anesthesia	−0.012	0.942
Residency level	R1	0.030	0.826
	R2	−0.048	0.703
	R3	−0.079	0.613
	R4	−0.042	0.754
	R5	0.158	0.800
Number of on-calls in the past month	No on-calls	0.015	0.927
	1–4	−0.012	0.903
	5–6	0.189	0.197
	7 or more	−0.184	0.495
Shift work	Yes	−0.194	0.398
Workload during the past month compared to usual workload	Less than usual	−0.013	0.910
	Unaffected	−0.007	0.951
	More than usual	−0.028	0.819

## Data Availability

Data can be requested from the principal investigator.

## Appendix A. Post Hoc Analyses for Groups with Significant ANOVA Test

**Table A1:** Post hoc analysis of the differences in JSE means between residents’ specialties.

Group 1	Group 2	Difference between Groups’ Means	Std. Error	*p* Value ^†^
Medical	Emergency medicine and critical care	9.13	3.42	0.111
	Family medicine	−1.13	2.56	0.999
	Ob/Gyn	6.38	4.46	0.785
	Pediatrics	−6.25	2.97	0.353
	Radiology	19.71	7.36	0.109
	Surgical and anesthesia	11.11	3.22	0.012
Family medicine	Emergency and critical care	10.27	3.57	0.066
	Ob/Gyn	7.51	4.58	0.657
	Pediatrics	−5.11	3.14	0.664
	Radiology	20.84	7.43	0.079
	Surgical and anesthesia	12.24	3.38	0.007
Pediatrics	Emergency and critical care	15.38	3.87	0.002
	Ob/Gyn	12.62	4.82	0.125
	Radiology	25.96	7.58	0.013
	Surgical and anesthesia	17.35	3.70	<0.001
Surgical and anesthesia	Emergency and critical care	−1.97	4.07	0.999
	Ob/Gyn	−4.73	4.98	0.964
	Pediatrics	8.60	7.69	0.922
Emergency and critical care	Ob/Gyn	−2.76	5.11	0.998
	Radiology	10.58	7.77	0.822
Ob/Gyn	Radiology	13.33	8.28	0.676

**^†^** Tukey’s multiple-comparisons test.

**Table A2:** Post hoc analysis of the differences in mean JSE scores between residents’ current rotations.

Group 1	Group 2	Difference between Groups’ Means	Std. Error	*p* Value ^†^
Medical	Emergency medicine and critical care	3.03	3.17	0.963
	Family medicine	−1.74	3.71	0.999
	Ob/Gyn	6.49	3.96	0.658
	Pediatrics	−8.19	3.06	0.110
	Radiology	5.21	5.51	0.965
	Surgical and anesthesia	6.04	2.94	0.384
Family medicine	Emergency and critical care	4.77	4.30	0.925
	Ob/Gyn	8.23	4.91	0.633
	Pediatrics	−6.45	4.22	0.727
	Radiology	6.95	6.23	0.923
	Surgical and anesthesia	7.78	4.13	0.494
Pediatrics	Emergency and critical care	11.22	3.76	0.048
	Ob/Gyn	14.68	4.44	0.019
	Radiology	13.40	5.87	0.256
	Surgical and anesthesia	14.23	3.56	0.002
Surgical and anesthesia	Emergency and critical care	−3.01	3.66	0.982
	Ob/Gyn	0.45	4.36	1.000
	Pediatrics	−0.83	5.81	1.000
Emergency and critical care	Ob/Gyn	3.46	4.52	0.988
	Radiology	2.18	5.92	1.000
Ob/Gyne	Radiology	−1.28	6.38	1.000

**^†^** Tukey’s multiple-comparisons test.

**Table A3:** Post hoc analysis of differences in JSE means between different residency levels.

Group 1	Group 2	Difference between Groups’ Means	Std. Error	*p* Value ^†^
R2	R1	2.08	2.72	0.941
	R3	0.91	2.96	0.998
	R4	−2.67	2.73	0.865
	R5	19.1	7.02	0.054
R1	R3	−1.17	3.04	0.995
	R4	−4.75	2.82	0.447
	R5	17.02	7.05	0.115
R4	R3	3.58	3.06	0.768
	R5	21.77	7.05	0.019
R3	R5	18.19	7.15	0.084

**^†^** Tukey’s multiple-comparisons test.

**Table A4:** Post hoc analysis of differences in mean JSE score by number of on-calls.

Group 1	Group 2	Difference between Groups’ Means	Std. Error	*p* Value ^†^
1–4 on-calls	No on-calls	3.11	2.75	0.669
	5–6 on-calls	2.69195	2.59948	0.729
	7 or more on-calls	11.23	4	0.028
5–6 on-calls	No on-calls	0.42	3.16315	0.999
	7 or more on-calls	8.54	4.29384	0.195
No on-calls	7 or more on-calls	8.12043	4.38452	0.252

**^†^** Tukey’s multiple comparisons test.
